# Insights into the Electronic Structure of a U(IV) Amido and U(V) Imido Complex

**DOI:** 10.1002/chem.202200119

**Published:** 2022-03-14

**Authors:** Luisa Köhler, Michael Patzschke, Stephen Bauters, Tonya Vitova, Sergei M. Butorin, Kristina O. Kvashnina, Moritz Schmidt, Thorsten Stumpf, Juliane März

**Affiliations:** ^1^ Helmholtz-Zentrum Dresden-Rossendorf (HZDR) Institute of Resource Ecology Bautzner Landstraße 400 01328 Dresden Germany; ^2^ The Rossendorf Beamline at ESRF at the European Synchrotron, CS40220 38043 Grenoble Cedex 9 France; ^3^ Karlsruhe Institute of Technology Institute for Nuclear Waste Disposal (INE) P.O. Box 3640 76021 Karlsruhe Germany; ^4^ Condensed Matter Physics of Energy Materials X-ray Photon Science Department of Physics and Astronomy Uppsala University P.O. Box 516 SE-751 20 Uppsala Sweden

**Keywords:** carbenes, electronic structure, HERFD XANES, ITI, uranium(V)

## Abstract

Reaction of the *N*‐heterocylic carbene ligand ^i^PrIm (L^1^) and lithium bis(trimethylsilyl)amide (TMSA) as a base with UCl_4_ resulted in U(IV) and U(V) complexes. Uranium's +V oxidation state in (HL^1^)_2_[U(V)(TMSI)Cl_5_] (TMSI=trimethylsilylimido) (**2**) was confirmed by HERFD‐XANES measurements. Solid state characterization by SC‐XRD and geometry optimisation of [U(IV)(L^1^)_2_(TMSA)Cl_3_] (**1**) indicated a silylamido ligand mediated inverse *trans* influence (ITI). The ITI was examined regarding different metal oxidation states and was compared to transition metal analogues by theoretical calculations.

## Graphical abstract

**In the compound** [U(V)(TMSI)Cl_5_], uranium could be stabilized in the rare +V oxidation state proven by HERFD XANES measurements. In the complex, an inverse *trans* influence (ITI) was uncovered. Quantum chemical calculations show this effect to be dependent on the electronic situation on the metal centre, thus increasing with higher oxidation state.

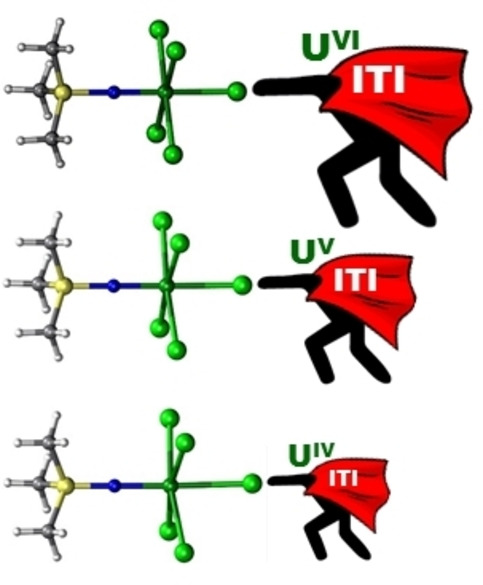

## Introduction

For years, chemists have strived to solve the persistent question of how the 5*f*‐electrons participate in and influence chemical bonding. Especially for the early actinides Th−Am the 5*f*‐orbitals are rather diffuse, and near degeneracy of 5*f‐*, 6*d‐*, and 7*s*‐orbitals enables a rich chemistry.[^1^, ^2^, ^3^, ^4^] As a result, some of these elements can access oxidation states of +II to +VII under certain conditions.[^3^, ^5^, ^6^, ^7^, ^8^, ^9^] Even for the extensively studied element uranium, the stabilization of its more exotic oxidation states still finds widespread interest. This concerns in particular the low oxidation states +II to +IV,^[10]^ of which U(II) was stabilized in solution only in 2013 by Evans et al.,^[6]^ but also U(V). The latter exhibits a strong tendency to disproportionate, indicating its instability in both the ‐yl and pure form. However, the Mazzanti group could identify a water stable U(V) complex^[11]^ and Liddle et al. were able to find U(V) also as a disproportionation product recently.^[12]^ The main factors influencing the stability of a certain oxidation state and thus reactivity arise from differences of the actinides’ electronic structure, which is strongly affected by for example organic ligands as complexation partners but also by the reaction conditions. In recent years, *N* donor ligands gained centre stage for stabilizing uranium over a wide range of oxidation states due to the quite flexible number of bonds they can form: They can thus be utilized as amide[^12^, ^13^, ^14^, ^15^] or imine[^16^, ^17^, ^18^, ^19^] functionalities, and even triple bonds as nitrides[^12^, ^20^, ^21^, ^22^, ^23^] are possible. Interestingly, bonds from imino ligands to U(VI) are more covalent compared to their dioxo analogues and capable of inducing further electronic effects like the inverse *trans* influence (ITI), opening an opportunity to shed light on 5*f*‐orbital involvement.^[24]^

In contrast to the existing *trans* influence in transition metal‐ and 4*f*‐element compounds,[^25^, ^26^] the ITI is a phenomenon in actinide chemistry due to the more diffuse 5*f*‐orbitals being in the valence space. It has predominantly been reported for actinides in high oxidation states (V, VI).[^27^, ^28^, ^29^] In the past, an ITI was identified in several U(V) and U(VI) complexes, with a strong bias towards the much more stable U(VI).[^28^, ^29^, ^30^, ^31^, ^32^, ^33^, ^34^, ^35^] Gregson et al. recently used carbenes as soft ligands to synthesise the first examples of An(IV) complexes exhibiting an ITI, all containing linear C=M(IV)=C (M(IV)=Th, U) entities built up by coordination to BIPM^TMS^ (BIPM^TMS^={C(Ph_2_PNSiMe_3_)_2_}^2−^) ligands.^[36]^ In the same study a Ce(IV) analogue was also reported, establishing the ITI as a general *f*‐block, rather than an actinide, concept. The ITI is expressed through remarkably short bonds of *trans* residing ligands, often involving strong donors, like oxo, imido, and nitrido ligands.[^27^, ^29^, ^36^] Although its origin is not fully understood, an orbital‐based approach to explain the ITI has been suggested by Denning.^[37]^ Strong donor ligands cause a quadrupolar charge distribution at the metal core through the interaction of filled 6*p* with vacant 5*f*‐orbitals, which leads to an accumulation of negative charge in the *cis* position of a strong donor. The ITI then arises from electrostatic repulsion between this negative charge and anionic ligands.[^24^, ^30^, ^38^, ^39^] More recently, the Liddle group proposed a different explanation, where strong ligands generate electrostatic potentials, which in the case of *d*‐orbital domination are *cis* directing through ionic interactions. If, however, short metal‐ligand bonds lead to stronger *f*‐orbital involvement, this ionic effect is overpowered and results in *trans* arrangement. In contrast to former ITI investigations, they show a contrary interaction of ionic and covalent effects in complexes with metals’ *f*‐orbitals, thus suggesting *f*‐orbital overlap‐driven covalency to act structure‐directing.^[40]^

Here, we report the synthesis of novel U(IV) and U(V) complexes containing TMSA (bis(trimethylsilyl)amido) and TMSI (trimethylsilylimido) ligands, respectively. Our aim is to not only expand the number of known structurally characterized U(IV/V) complexes but also to gain a deeper understanding of the compounds’ electronic structure and bond properties with the help of quantum chemical calculations and High Energy Resolution Fluorescence Detected X‐Ray Absorption Near Edge Spectroscopy (HERFD XANES). We will analyse and compare the bonding situation in both complexes and address the relative strength of the ITI as a function of the oxidation state of U, by comparison with other structure‐guiding effects, as well as a series of transition metal complexes exhibiting the *trans* influence (TI).

## Experimental Section

**Methods**: Infrared spectra were measured in solid state with an *Agilent Technologies Cary 630 FTIR*‐spectrometer, scanning a range of 4000–500 cm^−1^. For single crystal X‐ray diffraction analysis a *Bruker D8 Venture* diffractometer with a microfocus Mo‐K_*α*_‐ source (λ=0.71073 Å), a *Photon 100 CMOS* detector and *Oxford Cryostream* system were used. To avoid crystal damage through oxygen and moisture, the samples were selected under mineral oil via an optical microscope, mounted on a *MicroMount* ^*TM*^ (*MiTeGen*, USA) and placed into a continuous N_2_ flow. Data collection was performed through generic ϕ‐ and ω‐scans and integrated with Bruker *APEX 3* software with *SAINT* package. The multi‐scan method (*SADABS*) was used for empirical absorption correction. Structure solution was conducted via full‐matrix least‐squares method on F^2^ with Bruker *SHELXTL* package. All non‐hydrogen atoms were anisotropically refined, placed at calculated positions and allowed to ride on their parent atoms. X‐ray absorption near edge structure (XANES) in high energy resolution fluorescence detection (HERFD) mode at the U *M*_4_ edge was collected at CAT‐ACT beamline^[41]^ at the KIT Light Source, Karlsruhe Research Accelerator (KARA). The incident energy was selected using the <111> reflection from a double Si crystal monochromator. The U HERFD spectra at the *M*_4_ edge were obtained by recording at the maximum intensity of the U *M*β emission line (ca. 3339.8 eV) as a function of the incident energy. The emission energy was selected using the <220> reflection of one spherically bent Si crystal analyzer (with 1 m bending radius) aligned at 75° Bragg angle. Samples in form of crystalline powder were fixed on sticking tape and packed into specially designed holders consisting of two confinements with 8 and 25 μm Kapton foils as a 1^st^ and 2^nd^ confinement, respectively. The holders were then placed into an inert gas container for safe transport and measured under constant helium flow in order to avoid sample degradation.

**Quantumchemical Calculations**: All calculations were carried out with the Turbomole package (version 7.3.1).^[42]^ DFT calculations using the PBE0 functional were performed with a def‐TZVPP basis set for all elements.^[43]^ Relativistic effects were included via the use of ECPs for tungsten and uranium.[^44^, ^45^] Dispersion effects were considered with the approach according to Grimme and the D3 parameter set.^[46]^ The COSMO model with the dielectric constant set to infinity was used to simulate solid state structures.^[47]^ Further analysis of the calculated electron density was performed with the AIMALL program.^[48]^ Especially the localisation index (LI) and the delocalisation index (DI) were extracted.

The isotropic XANES spectrum was calculated in a manner described by Butorin et al. by taking into account the full multiplet structure due to intra‐atomic and crystal field interactions.^[49]^ The Slater integrals F^k^(5 *f*,5 *f*), F^k^(3*d*,5 *f*) and G^k^(3*d*,5 *f*) calculated for the U(V) ion were scaled down to 80 % of their *ab initio* Hartree‐Fock values. The ground and final states of the spectroscopic process were represented by the 3*d* ^10^5*f* ^1^ and 3*d* ^9^5*f* ^2^ configurations, respectively. The U(V) environment was approximated by D_4h_ symmetry. The Wybourne's crystal field parameters were set to B^2^_0_=0.50 eV, B^4^_0_=2.00 eV, B^4^_4_=−0.37 eV, B^6^_0_=−1.50 eV and B^6^_6_=−1.17 eV. This choice originates from Liu et al. where a set of parameters was derived for uranyl in D_4h_ symmetry for a few compounds by fitting their optical absorption data.^[50]^ The actual values of the parameters in our calculations were however somewhat increased as compared to those in Ref. [50] to compensate for omitting the effects of the 5*f* hybridization with ligand states which leads to some changes in the spread of the energy levels. Furthermore, the choice of the parameter values is facilitated by comparing the calculated energies of the multiplet states of the ground state configuration with those calculated using density functional theory.

**Synthetic Procedures**: *Caution! Natural uranium is consisting of radioactive nuclides including long‐lived α‐emitters (^235^U; T*_*1/2*_ *=7.04×10* ^*8*^ *years, and* ^*238*^ *U; T*_*1/2*_ *=4.47×10* ^*9*^ *years). Special precautions as well as appropriate equipment and facilities for radiation protection are required for handling this material. All experiments were carried out in a controlled laboratory at the Institute of Resource Ecology, Helmholtz‐Zentrum Dresden‐Rossendorf*.

All experiments were conducted in *M. Braun* gloveboxes with a N_2_ atmosphere. Solvents were distilled prior to use and stored under nitrogen over 3 Å molecular sieve. Acetonitrile (<10 ppm water content) was used without further purification, LiTMSA and ^i^Pr_2_ImHCl were used as received. UCl_4_ was synthesized according to literature.^[51]^

**[u(iv)( ^i^ pr_2_ im)_2_(tmsa)cl_3_ ] (1)**: ^i^Pr_2_ImHCl (55.1 mg, 0.3 mmol) and LiTMSA (64.0 mg, 0.4 mmol) were dissolved in 2 mL thf. After stirring at room temperature for 1 h, UCl_4_ (60.3 mg, 0.2 mmol) in 2 mL thf was added. Colour change occurred immediately from colourless to dark brown. After one day of stirring, the reaction mixture was centrifuged (145 rpm, 60 sec) and a brown, clear mother liquor was obtained. Diffusion experiments with benzene yielded green crystals, suitable for SC‐XRD. Yield: 28 %. IR [cm^−1^]: 2919 (vs), 2847 (s), 1550 (vw), 1460 (m), 1374 (m), 1203 (m), 1180 (w), 1144 (w), 1067 (vw), 751 (m).

**(HL^1^)_2_[U(V)(TMSI)Cl_5_] (2)**: The synthesis was done analogously to **1**. However, ^i^Pr_2_ImHCl (55.2 mg, 0.3 mmol) and LiTMSA (48.2 mg, 0.3 mmol) were dissolved in 2 mL acetonitrile. Then, a suspension of UCl_4_ (55.2 mg, 0.15 mmol) in 1 mL acetonitrile was added. Stirring for several hours at room temperature and subsequent work up led to a brown mother liquor. Diffusion experiments with benzene yielded green platelet crystals. Yield: 15 %. IR [cm^−1^]: 3130 (w), 2980 (w), 1640 (vw), 1551 (m), 1455 (m), 1380 (m), 1325 (vw), 1270 (m), 1236 (s), 1181 (s), 1140 (s), 1004 (w), 955 (w), 901 (vs), 833 (vs), 743 (s).

Deposition Numbers 2017687 (for **1**) and 2017686 (for **2**) contain the supplementary crystallographic data for this paper. These data are provided free of charge by the joint Cambridge Crystallographic Data Centre and Fachinformationszentrum Karlsruhe Access Structures service.

## Results and Discussion

**Figure 1:**
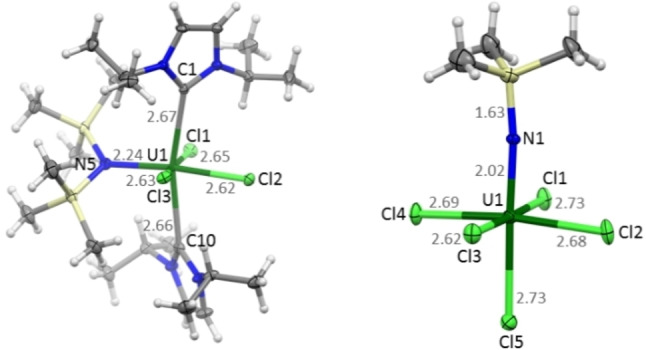
Molecular structures of [U^IV^(L^1^)_2_(TMSA)Cl_3_] (**1**, left) and [U^V^(TMSI)Cl_5_]^2−^ (**2**, right) from SC‐XRD. Counter ions and solvent molecules omitted for clarity. Colour code: carbon (C, gray), chlorine (Cl, lime), hydrogen (H, white), nitrogen (N, blue), silicon (Si, yellow), and uranium (U, green).

SC‐XRD investigations of **1** revealed a U(IV) complex with the metal centre surrounded by two carbenes, one TMSA and three chloro ligands in a distorted octahedral coordination geometry around the uranium (see Figure 1). The chloro and the TMSA ligands are arranged equatorially around the metal centre, whereas the carbenes occupy axial positions. Due to the bulky TMSA ligand, the C1‐U1‐C10 angle is reduced to 166°, compared to the expected 180°. Furthermore, the carbenes form a dihedral angle of 89° to avoid steric repulsion between its isopropyl groups and the trimethylsilyl residues of the TMSA ligand. This is also the reason for the latter being shifted out of the C‐U‐C plane by 57°. The coordinating carbene ligands exhibit U−C bond distances of 2.657(2) Å and 2.667(2) Å, lying in the range of long U(IV)−C single bonds. This can be interpreted as a relatively weak, dative interaction.^[52]^ Among the U−Cl bond lengths, U1‐Cl2 is 2.617(1) Å and thus 0.013 Å and 0.033 Å shorter than the other U−Cl distances (2.630(1) Å for U1−Cl1 and 2.650(1) Å for U1−Cl3), which may be a first indication of additional effects acting on this bond. These effects become obvious considering the remarkably short bond distance to the TMSA ligand U1−N5 in *trans* position to the shortest U−Cl bond, which is only 2.242(2) Å. This is significantly shorter than in the [U(N(SiMe_3_)_2_)_4_] complex (2.294 Å–2.301 Å),^[53]^ but still in the expected range for U−N single bonds.^[53]^ Thus, both bonds U1−Cl2 and U1−N5 are shortened in **1**, pointing to an ITI for those ligands. To the best of our knowledge, this is the first example of an amido ligand being responsible for an inverse *trans* influence, which is typically reserved for much stronger donors capable of forming multiple bonds to the metal centre, such as imido, oxo, or nitrido ligands.[^28^, ^29^, ^35^, ^36^] This might be an explanation why there is only a relatively small shortening of the mentioned *trans* bonds. The TMSA ligand has been used previously in investigations of the ITI in U(V) and (VI) compounds, however, it was always employed as a co‐ligand in these systems and not as the ligand inducing the effect.[^13^, ^34^]

Unexpectedly, when changing the solvent to acetonitrile, we obtained compound **2** as product. SC‐XRD investigations of **2** show that uranium is coordinated by one trimethylsilylimido (TMSI) and five chloro ligands, leading to an octahedral complex dianion (see Figure 1). Two additional imidazolium cations are present, which indicates a charge of −2 for the complex anion. The U1−N1 bond length is very short at only 2.019(9) Å, in agreement with a U=N double bond, which can for example be found in a U imido complex by the Meyer group.[^54^, ^55^] This interpretation can be confirmed by quantum chemical calculations, which find a *Mayer* bond order of 1.8. Traces of water in the acetonitrile could be responsible for the protonation of ^*i*^PrIm in a first step, and could further serve as an oxidant for U(IV) to U(V), combined with a rearrangement of the TMSA ligand, forming the TMSI anion (a suggestion for a reaction mechanism is given in the Supporting Information, chapter S5). IR spectroscopic data point to a pronounced blueshift of the characteristic U−N stretching vibration from ∼750 cm^−1^ in **1** to ∼1000 cm^−1^ in **2** for the U=N double bond (see also chapter S2 in the Supporting Information).

**Figure 2:**
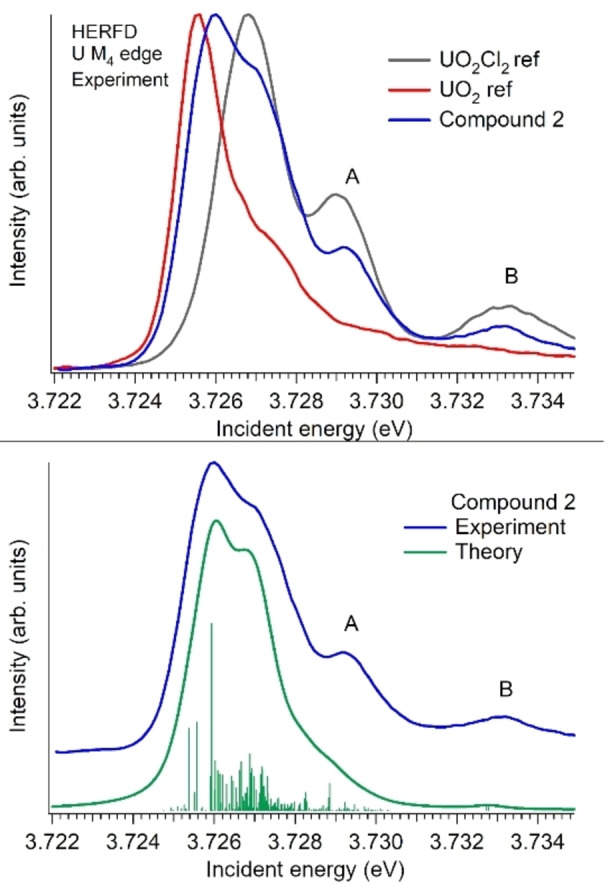
Adjusted HERFD data, where the background was substracted by the Savitzky‐Golay Filtering option in the PyMCA program (top panel) and theoretical calculations, using the Anderson Impurity Model (AIM) approximation (bottom panel).

To confirm the oxidation state of U and concurrently obtain additional insights into its electronic structure, HERFD XANES measurements were performed.[^54^, ^56^, ^57^] In Figure 2 we show the HERFD data taken at the U *M*_4_ edge (∼3.728 keV) with the emission spectrometer tuned to maximum of the *Mβ* line (3.337 keV). At the U *M*_4_ edge, we probe the transitions from the ground electron shell 3*d* ^10^4*f* ^14^5*f* ^*n*^ to the 3*d* ^9^4*f* ^14^5*f* ^(*n*+1)^ shell in the U atom, and at the same time we record the event when the electrons from the core occupied shells (4*f*) fill the created hole at the ground states 3*d* ^9^ 4*f* ^14^ 5*f* ^(*n*+1)^ to 3*d* ^10^ 4*f* ^13^ 5*f* ^(*n*+1)^. Due to the dipole selection rules (*J*=0; ±1) the unoccupied 5*f*_5*/*2_ electronic level can be reached at the U *M*_4_ edge from the 3*d*_3*/*2_ state. Thus, U *M*_4_ HERFD can probe the number of the 5 *f* electrons at the ground state i. e. the oxidation state of U. Compound **2** in U(V) configuration should have *5f* ^*1*^ ground state configuration, while UO_2_ with U(IV) oxidation state has *5f* ^*2*^ configuration and UO_2_Cl_2_ with U(VI) oxidation states has *5f* ^*0*^ configuration.[^56^, ^58^, ^59^, ^60^]

The first noticeable difference between the HERFD spectra of **2** and the reference compounds (UO_2_ and UO_2_Cl_2_) is the shift of the white line in the incident energy, which indicates the presence of the U(V) oxidation state. Moreover, we noticed, that Zegke et al. recently reported the HERFD spectrum of a U(V)‐uranyl compound and the shape of its HERFD spectrum is very close to the one recorded on compound **2**.^[61]^ However, the HERFD spectrum of the compound reveals additional structures, marked A and B in Figure 2. These cannot be reproduced by our calculations and indicate the presence of small impurities of U(VI) in uranyl coordination in compound **2**. In order to confirm the assignment of the spectral features, we performed theoretical calculations of the U(V) *M*_4_ edge spectrum using crystal field theory (see Figure 2).

**Figure 3:**
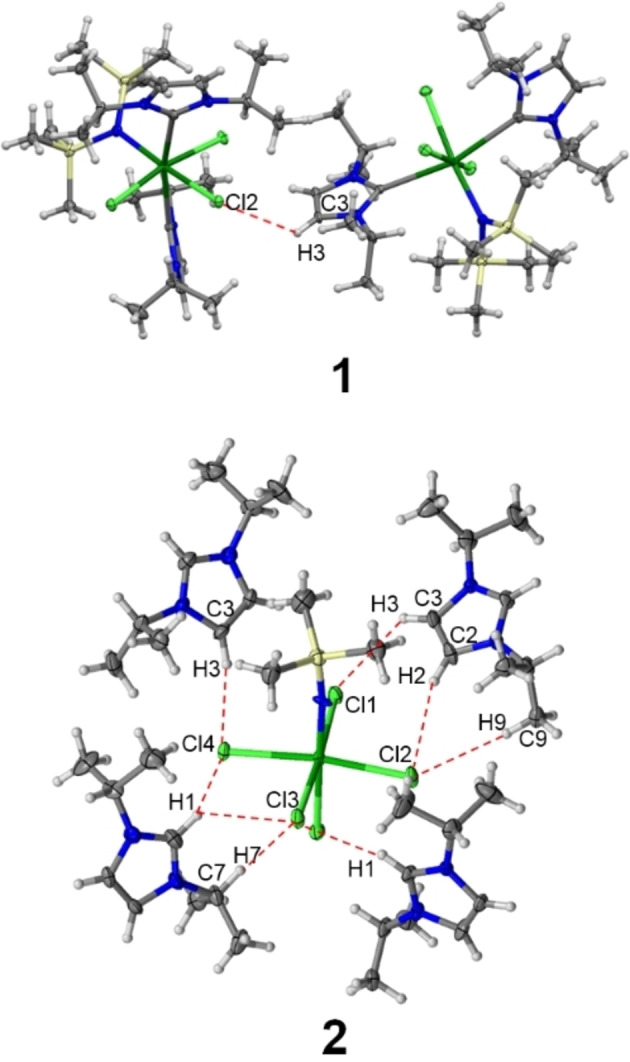
Hydrogen bonds in **1** (top) and **2** (bottom). Colour code: carbon (C, gray), chlorine (Cl, lime green), hydrogen (H, white), nitrogen (N, blue), silicon (Si, yellow), and uranium (U, green).

Our experimental data together with theoretical calculations confirm the presence of the predominant U(V) oxidation state.[^58^, ^59^, ^60^, ^61^, ^62^, ^63^, ^64^] Compound **2** is then only the fourth anionic non‐oxygen containing U(V) complex reported in the literature. In all other examples of this class of compounds the U(V) is stabilised through very large and bulky ligands, for example Tren^TIPS^ (Tren^TIPS^=N(CH_2_CH_2_NSi^i^Pr_3_)_3_) or dbabh^−^ (Hdbabh=2,3 : 5,6‐dibenzo‐7‐azabicyclo‐[2.2.1]hepta‐2,5‐diene) ligand.[^21^, ^23^, ^65^] In contrast, **2** is stabilized by the significantly smaller TMSI and chloro ligands. Within the TMSI ligand, the U1‐N1‐Si1 arrangement is almost linear (172°). The chlorine atom in *trans* position to the TMSI ligand possesses a U1−Cl5 bond length of 2.728(3) Å and is thus in the same range as the remaining four U−Cl distances (2.619(3) Å–2.726(3) Å). Consequently, the experimental data show no ITI in **2**, although it would be expected to be even stronger than in **1**. This assumption is based on 1) the higher charge of the metal centre and 2) the more strongly donating imido ligand relative to the amido ligand in **1**, forming a U1=N1 double bond.^[66]^ A possible explanation for the absence of the ITI can be found in the molecular packing of **2** which exhibits numerous bi‐ and trifurcated hydrogen bonds C−H⋅⋅⋅Cl as well as weak electrostatic interactions between the complex anion and the imidazolium cations (see Figure 3, bottom).

The C−H⋅⋅⋅Cl interactions range between 2.62 Å and 2.90 Å (H⋅⋅⋅Cl) with angles (C−H⋅⋅⋅Cl) between 128° and 168°. Every chloro ligand is involved in hydrogen bonding to either one or two imidazolium cations, as part of a strong hydrogen bond network within the crystal structure of **2**.^[67]^ In contrast, the intermolecular interactions in **1** are limited to one weak hydrogen bond C3−H3⋅⋅⋅Cl2 and a Cl⋅⋅⋅π interaction involving the carbene ligand (see Figure 3, top). A summary of all hydrogen bonding parameters of **1** and **2** can be found in the Supporting Information.

**Figure 4:**
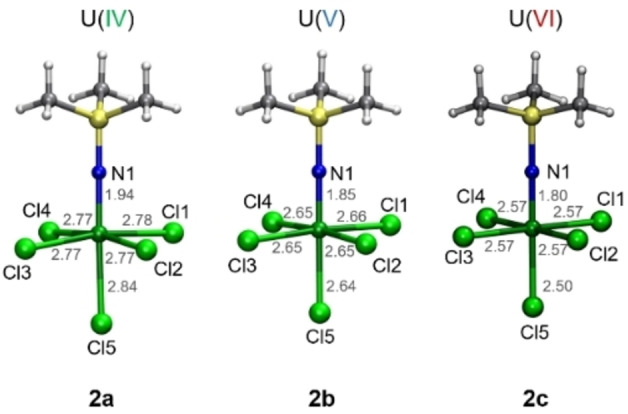
Optimised structures of [U^IV^(TMSI)Cl_5_]^3−^ (**2 a**), [U^V^(TMSI)Cl_5_]^2−^ (**2 b**) and [U^VI^(TMSI)Cl_5_]^−^ (**2 c**). Colour code: carbon (C, gray), chlorine (Cl, lime), hydrogen (H, white), nitrogen (N, blue), silicon (Si, yellow), and uranium (U, green).

In order to disentangle the ITI from these additional effects and to elucidate the influence of uranium's oxidation states on the ITI, structure optimisations of **2** and its hypothetical analogues containing U(IV) and U(VI) were performed. Initial unbiased optimisations lead to U‐N‐Si angles deviating from the experimental values, which cause additional interactions of the methyl groups of TMSI with the chlorine atoms. In order to avoid such additional interactions and more accurately reproduce the experimentally determined structure, optimisations were performed in which the U‐N‐Si angle was kept at 180°, and these structures are shown in Figure 4.

As expected, bond lengths to uranium decrease with increasing oxidation state. The contraction is, however, anisotropic and does not systematically follow the cation's ionic radii [r_ion_(U(IV))=0.89 Å, (U(V))=0.76 Å, (U(VI))=0.73 Å].^[68]^ To investigate the ITI in these structures, we focus on the U1−Cl5 and U1−N1 bonds. In the U(VI) compound the former bond is 0.07 Å shorter than those to the equatorial chloro ligands. For U(V), this difference decreases to 0.01 Å and for U(IV) the bond to the axial chloro ligand is longer by 0.07 Å. This interesting trend can be explained by a combination of two counteracting effects. Firstly, the ITI decreases for lower oxidation states. Secondly, the lower oxidation states exhibit lower charges at the metal centre, which increases Coulomb repulsion between the axial and equatorial chloro ligands. For the U(IV) complex, even the strong ITI of the TMSI ligand is not strong enough to counteract this Coulomb repulsion. The calculated U−Cl bond lengths are more even but on average of the same length as in the XRD structure. However, the calculated U−N bond length is about 0.15 Å shorter than the experimentally determined one. The large deviations are due to the extended hydrogen network in crystal **2**, which cannot be reproduced in our calculations. The chloro ligand residing *trans* to TMSI experiences its strongest bond shortening in the U(VI) complex, compared to the decrease of U's ion size U(VI)−Cl5 is shortened vs. U(V)−Cl5 by 0.14 Å, which is 0.09 Å more than the decrease of the ionic radii. A similar effect of 0.07 Å excess shortening can be found for U(V)−Cl5 vs. U(IV)−Cl5. In summary, all compounds do exhibit an ITI with the expected strength sequence VI>V>IV. However, the complex geometry, inter‐ and intramolecular interactions may rapidly overcompensate the inverse *trans* influence.

To improve our understanding of the origin of the ITI, the strength of the ITI in these *f*‐element complexes ought to be compared to a similar structure of a transition metal complex showing a *trans* influence (TI). This is a well‐known bond lengthening of *trans* residing ligands found in transition metal complexes. To this end, structures analogue to **2 a**–**2 c** were optimised with a tungsten centre in the same oxidation states +IV to +VI (see Supporting Information). The picture that emerges is quite interesting. The oxidation state of the metal centre has very little influence on the magnitude of the TI. For all three oxidation states, the bond to Cl5 is 0.22–0.24 Å longer than those to Cl1‐4, indicating a significant weakening of the bond to the axial chloro ligand. The fact that we go from a bond lengthening of more than 0.2 Å for the transition metal to a bond shortening for the actinide is a clear indication of the strength of the TMSI ligand. To understand the bonding situation in the two series, electronic properties like NBO charges of the metal centres q(M)NBO and delocalisation indices (DI) for all bonds involving the metal have been collected in Table 1. The DI is a measure of the number of electrons shared between two atoms and is related to the Wiberg bond order.[^69^, ^70^] We also give a computational measure for the oxidation state (OS) of the metal centre, defined as the difference of nuclear charge Z and localisation index λ. Considering a mostly ionic bonding situation, the localisation index gives a measure of the numbers of electrons remaining at the metal centre. Subtracting this number from the nuclear charge should provide a number comparable to the oxidation state of that metal.^[71]^

**Table 1:** QTAIM parameters and NBO charges for the W and U compounds. For the definition of “OS” please refer to the text.

	W(IV)	W(V)	W(VI)	U(IV)	U(V)	U(VI)
q(M)NBO	0.95	1.04	1.00	1.11	0.75	0.43
“OS” Z(M)‐λ(M)	4.27	4.79	5.29	4.32	5.14	5.85
DI(M−N)	1.67	2.00	2.06	1.60	1.95	2.20
DI(M−Cl_eq_)	0.59	0.63	0.73	0.44	0.59	0.77
DI(M−Cl_ax_)	0.34	0.33	0.39	0.42	0.68	0.93

From these data it becomes clear, that the oxidation states of the calculated species are as chemically assumed. For tungsten, the results are somewhat lower than expected, which is due to the long axial chloro bond leading to a slightly lower than expected chemical oxidation state. The tungsten charges do not vary much, the higher charge one would expect for the higher oxidation state is counterbalanced by the closeness of the equatorial chloro ligands, which leads to improved backbonding. For uranium on the other hand, the calculated oxidation states agree very well with the expected values. The uranium charges, however, actually go against this trend with U(VI) having the lowest charge. This unusual charge trend can be explained based on the same process that induces the ITI in the uranium compounds: the electron hole in U 6*p* is filled by the strong ligand, thereby lowering the charge on uranium. In addition, the axial chlorine binds very strongly to the uranium, further lowering its charge (see S6 in Supporting Information for further information).

## Conclusion

We synthesised a novel U(IV) *bis*‐carbene complex **1**, which exhibits a more pronounced ITI than expected for U in this low oxidation state. Furthermore, uranium could be stabilized in its elusive +V oxidation state in a rare dianionic TMSI complex **2**. Here, the ITI is not experimentally observable, even though its presence was confirmed in quantum chemical calculations. A quantification of the ITI is difficult because of a lack of an ITI/TI innocent reference to compare to and because of the interplay of several effects, like repulsion or interactions between Cl ligands. However, we could confirm that the ITI is overcompensated by a network of intra‐ and intermolecular interactions in the SC‐XRD structure. Theoretical investigations on the influence of the metal oxidation state on the ITI were performed considering U(IV/V/VI) in the molecular structure of **2**. The results demonstrate a stronger ITI with increasing oxidation state. On the contrary, for a calculated series of W(IV/V/VI) complexes a strong TI independent of the metal's oxidation state was found. The strength of the ITI in **2** was evaluated by comparing the U(V) complex to its W(V) analogue. In general, the ITI is weaker than the TI, but still distinct. Our results expand the limited number of uranium compounds, especially in the rare +V oxidation state, and shed light on its electronic bond properties.

## Conflict of interest

The authors declare no conflict of interest.

1

## Supporting information

As a service to our authors and readers, this journal provides supporting information supplied by the authors. Such materials are peer reviewed and may be re‐organized for online delivery, but are not copy‐edited or typeset. Technical support issues arising from supporting information (other than missing files) should be addressed to the authors.

Supporting Information

## Data Availability

The data that support the findings of this study are available in the supplementary material of this article.
