# Supplementary material for: Insights into the Electronic Structure of a U(IV) Amido and U(V) Imido Complex
Source: Chemistry. 2022 Mar 14;28(21):e202200119. doi: 10.1002/chem.202200119 (PMC9310906; doi:10.1002/chem.202200119)
Supplement: Supplementary file 1 — Supporting Information [file CHEM-28-0-s001.pdf]

Chemistry–A European Journal

Supporting Information

Insights into the Electronic Structure of a U(IV) Amido and U(V) Imido Complex

Luisa Köhler, Michael Patzschke, Stephen Bauters, Tonya Vitova, Sergei M. Butorin, Kristina O. Kvashnina, Moritz Schmidt, Thorsten Stumpf, and Juliane März*

Table of Contents

S1 NMR Spectroscopy 2
S2 IR Spectroscopy 2
S3 Crystallographic Data and Bond Lengths 4
S4 Intermolecular Interactions 6
S5 Suggested Mechanism for the Formation of Complex **2** 6
S6 NBO Charges and the ITI 6
S7 References 7

S1 NMR Spectroscopy

NMR investigations could not be performed, because of the low complex concentration in solution. Increasing concentration by collecting several crops was not successful, because of the instability of the title compounds.

S2 IR Spectroscopy

Through the DFT based structure optimizations of complexes **1** and **2**, their vibrational frequencies can be obtained. To facilitate comparison of computational (harmonic) and experimental frequencies, the former have been scaled by a factor of 0.95 as suggested by Truhlar et al.[1] As shown in Figure 1 the experimental and calculated spectra are in good agreement. Thus, the theoretical data can be used for assigning the signals to a vibration. These are shown in the tables 1 and 2 below with an additional comment where necessary. The most important bands regarding complexes **1** and **2** are connected to the U–N (for **1**) and U=N (for **2**) stretching vibrations, since they should be present at different wavenumbers. This provides the possibility to distinguish between the two compounds. Indeed, such bands could be found at around 750 cm^{-1} for the U–N stretching vibration and around 1000 cm^{-1} for the U=N vibration. These lie in the expected range for uranium amido[2] and metal imido stretching vibrations[3–5]. If the calculated vibrational modes are transformed into local modes, the difference in the U-N vibrational frequencies becomes even more pronounced. In such a basis, the local U-N mode changes from 701 cm^{-1} for **1** to 1061 cm^{-1} for **2**. This difference of around 360 cm^{-1} for the stretching vibrations of a U–N single and U=N double bond is in a similar range as for C–C single and C=C double bonds (~300 cm^{-1}; ~1300 $^{-1}$ for C–C and ~1600 $^{-1}$ for C=C).

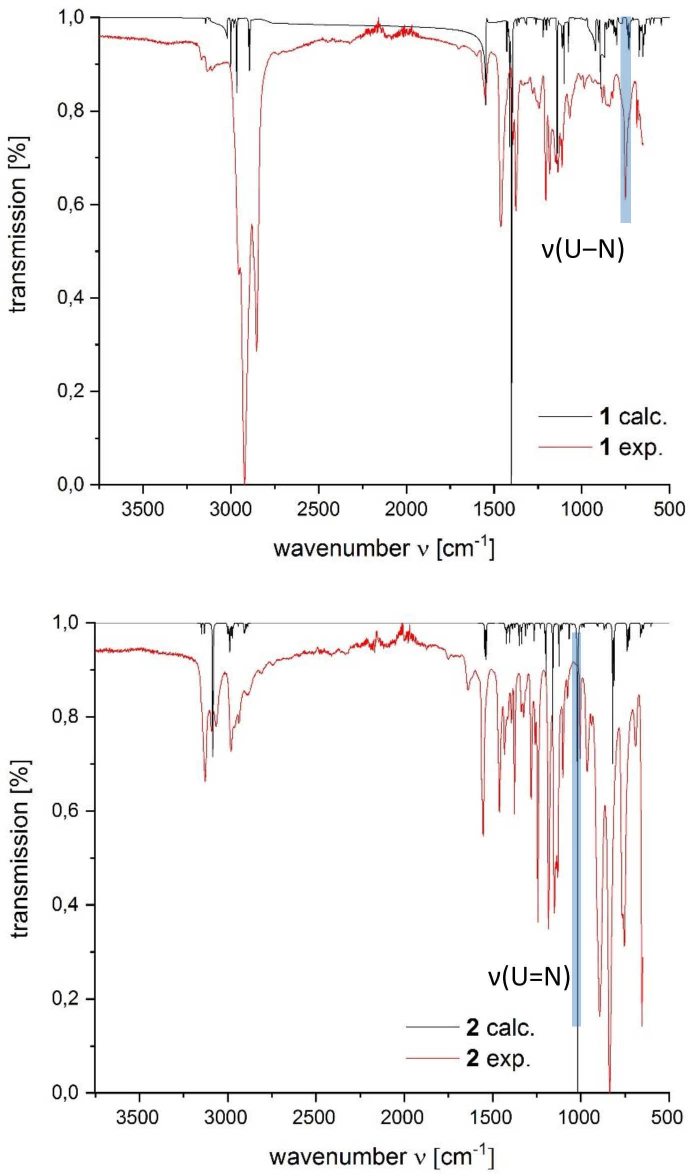

Figure 1 IR spectra of the complexes **1** and **2** (red) and the calculated spectra (black).

Table 1 Bands of the experimental IR spectrum of complex **1** and their assignment based on the calculated spectrum.

**complex 1**		
**ν [cm^{-1}]**	**assignment**	**comment**
2919	ν(HC=CH)<br>ν_{as}(HC=CH)	C-H stretching vibration of C=C entity in the carbene
2847	ν(CH_2)<br>ν(CH_3)	stretching vibration of CH_2 and CH_3 groups in the carbene
1550	ν(C=C)	stretching vibration of the C=C entity of the carbene
1460	δ(CH_3)	
1374	δ(CH_2)	
1203	δ(CH_3)	deformation vibration of the CH_3 groups in the TMSA ligand
1180	δ(C-H)	Wagging of the HC=CH group of the carbene
1144	δ(^{i}Pr)	deformation vibrations of the whole isopropyl group in the carbene
1067	δ(CH=CH)	
837	δ(U-N)	deformation vibration of the TMSA ligand, involving wagging of U-N bond
751	ν(U-N)<br>δ(CH_3)	U-N stretching vibration in combination with deformation vibration of TMSA methyl groups (broad band)

Table 2 Bands of the experimental IR spectrum of complex **2** and their assignment based on the calculated spectrum.

**complex 2**		
**ν [cm^{-1}]**	**assignment**	**comment**
3130	ν_{as}(CH_3)<br>ν(CH)	
2980	ν(CH_3),	
1640		
1551	ν(C-N)	skeleton vibrations of the carbene ring
1455	δ(CH_3) ^{i}PrIm	twisting deformation vibration of the isopropyl group of the carbene
1380	δ(CH_3)	
1325	δ(CH_2)	
1270	δ(CH_2)	
1236		
1181	δ(CH_3) TMSI	deformation vibration of the CH_3 groups in the TMSI ligand
1153	δ(CH) iPrIm	wagging vibration of the C=C entity in the carbenes
1100	various skeleton vibrations in the carbene	
1004	ν(U=N)	stretching vibration of N between U and Si
955		
833	δ(CH_3) TMS	deformation vibrations of the CH_3 groups in the TMSI Ligand
743	δ(CH) ^{i}PrIm<br>δ(CH_3) TMSI	
680	δ(CH_3)	deformation vibrations of all methyl groups

S3 Crystallographic Data and Bond Lengths

Table 3 Crystal structure data and refinement details for the reported structures **1** and **2**.

	**1**	**2**
**chemical formula**	$C_{27}H_{53}Cl_3N_5Si_2U$	$C_{21}H_{43}Cl_5N_5SiU$
**CCDC**	2017687	2017686
**M (g/mol)**	848.3.2	809.0
**crystal system**	monoclinic	monoclinic
**space group**	$P2_1/c$	$P2_1$
a **(Å)**	17.1956(7)	10.3650(4)
b **(Å)**	11.7769(5)	14.8857(6)
c **(Å)**	17.7744(8)	10.9168(4)
α **(°)**	90	90
β **(°)**	90.802(1)	94.603(2)
γ **(°)**	90	90
V **(Å³)**	3599.2(3)	1678.92(11)
T **(K)**	102	100
Z	4	2
**$\rho_{calc.}$ (mg/m³)**	1.566	1.600
**absorption coeff. (mm^{-1})**	4.82	5.29
**Θ_{max} (°)**	26.7	24.4
**R [$I > 2\sigma(I)$]**[a]	0.021	0.0334
**wR2(int)**[a]	0.0497	0.0888
w **scheme** ***d, e***	0.0193, 6.3353[a]	0.0240, 13.8315[a]
**data/param**	7635/357	3381/279
**res. dens (eÅ^{-3})**	1.61, −1.51	1.53, −2.56
**R_{int}**	0.057	0.03
**GooF**	1.07	1.12

[a] $w=1/[\sigma^2(F_o^2)+(0.0240P)^2+13.8315P]$ where $P=(F_o^2+2F_c^2)/3'$

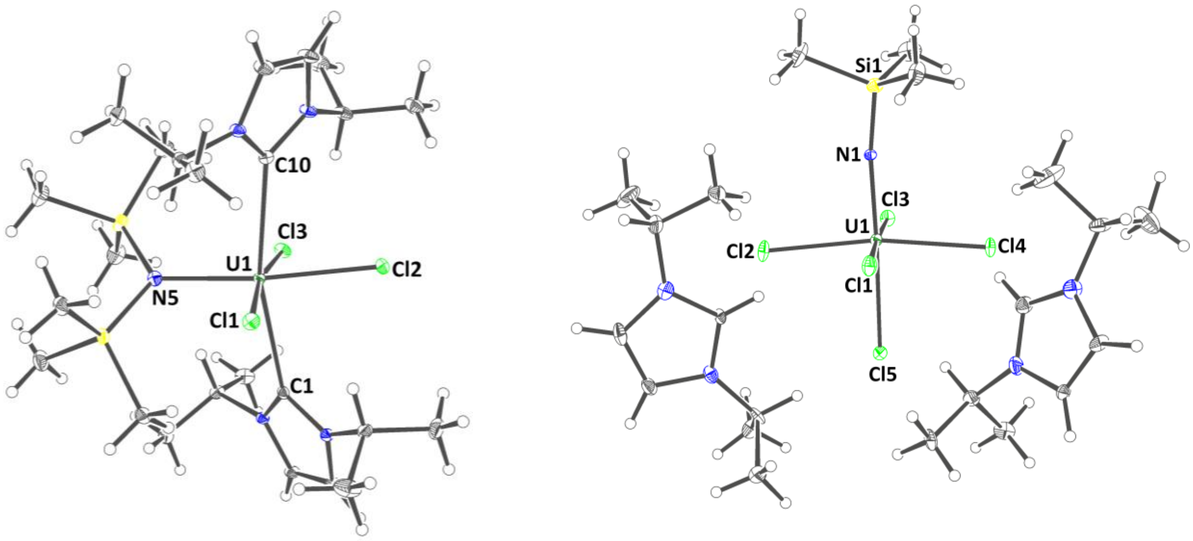

Figure 2: ORTEP plots of the compounds **1** (left) and **2** (right). Colour code: carbon (C, gray), chlorine (Cl, lime green), hydrogen (H, white), nitrogen (N, blue), silicon (Si, yellow), and uranium (U, green).

Table 4 Relevant bond lengths in complex **1** in Å.

atom 1	atom 2	d1,2 [Å]
U1	N5	2.242(2)
U1	Cl1	2.6299(7)
U1	Cl2	2.6166(8)
U1	Cl3	2.6500(6)
U1	C1	2.657(2)
U1	C10	2.667(2)

Table 5 Relevant bond lengths in complex **2** in Å.

atom 1	atom 2	d1,2 [Å]
U1	N1	2.019(9)
U1	Cl1	2.728(3)
U1	Cl2	2.678(3)
U1	Cl3	2.619(3)
U1	Cl4	2.689(3)
U1	Cl5	2.726(3)

S4 Intermolecular Interactions

Table 2 Intermolecular interactions in the reported crystal structures of **1** and **2**.

C	H	Cl	$d_{H,Cl}$ [Å]	$\angle_{C,H,Cl}$ [°]
**1**				
C3	H3	Cl2	2.85	118.0
C3		Cl2	3.40	
**2**				
C10	H10	Cl1	2.55	167.1
C4	H4	Cl1	2.88	139.1
C1	H1	Cl1	2.89	128.0
C15	H15B	Cl2	2.86	168.5
C12	H12	Cl2	2.73	160.0
C11	H11	Cl3	2.88	128.8
C3	H3	Cl4	2.63	157.9
C1	H1	Cl4	2.62	138.1
C14	H14	Cl5	2.90	137.2

S5 Suggested Mechanism for the Formation of Complex **2**

When repeating the synthesis of compound **1** in acetonitrile instead of thf, we have surprisingly received complex **2**. We suggest a two-step conversion of the U(IV)- into the U(V)-complex based on the assumption that traces of water are present in the acetonitrile solvent. In a first step, water could protonate the imidazolium-based carbene ligands to yield H^{i}PrIm cations. As a replacement, chloride ions still present from UCl_4 could coordinate to the uranium centre. Finally, homolytic bond breaking might lead to the electronic rearrangement including separation of the -$SiMe_3$ group.

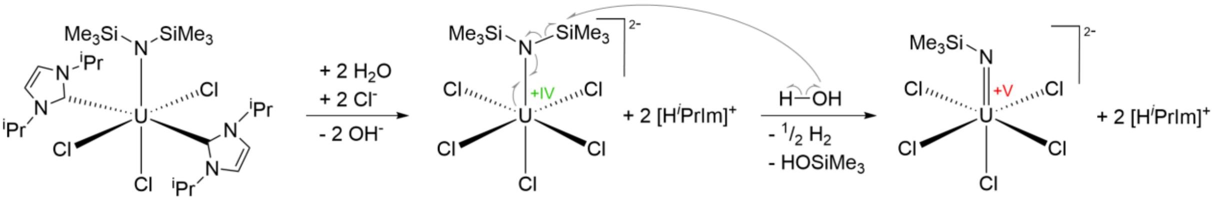

Figure 4 Mechanism suggestion for the transformation of the U(IV) complex **1** in the U(V) complex **2** leading to a double bonded imino ligand. The reaction was performed in acetonitrile with traces of water present in the solvent as an impurity.

S6 NBO Charges and the ITI

To gain further insight into the charge distribution in the U(V) imido system, further computations with a very weak ligand (BF_4^-) instead of TMSI were performed. Using the same level of theory we optimized the $[U^V(BF_4)Cl_5]^-$ system. For comparability we forced the B-F-Cl bond angle to be 180° as in the imido system. The first important result is, that the ITI all but vanishes. The difference in bond distances between the equatorial and axial chlorine bonds is reduced from 1.2 ppm for the imido system to 0.2 ppm for the tetrafluoroborate system. It is difficult to compare charges to prove the filling of the *p*-hole by the ligand, as there either is an ITI and a hole, which is then filled by electron donation from the *trans* standing ligand, or there is no ITI, in which case there is also no hole. The effect should however still be detectable by comparing the charges on the axial and equatorial chlorine atoms. These results can be found in Table 3. It is apparent that the charges are rather different, this is due to the higher total charge on the imido system. The important point is the charge difference between equatorial and axial chlorine atoms which is 0.07 in the system with ITI, but it is 0 for the system without ITI. This is due to electron transfer from the axial chlorine to the presumed electron hole for uranium in case of the imido system, as explained in the main text.

Table 3 NBO Charges for two U(V) species, one with, the other without ITI.

Atom	$[U^V(TMSI)Cl_5]^{2-}$	$[U^V(BF_4)Cl_5]^-$
U	0.75	0.41
Cl_{eq}	-0.45	-0.15
Cl_{ax}	-0.38	-0.15

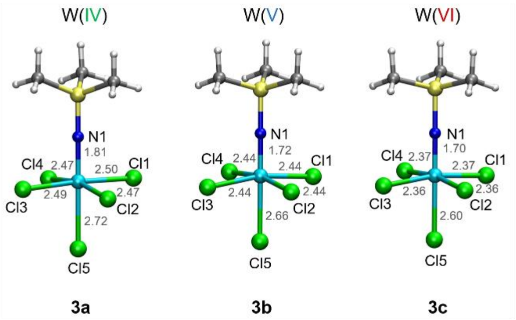

Figure 6 Optimized structures of $[W^{IV}(TMSI)Cl_5]^{3-}$ (**3a**), $[W^V(TMSI)Cl_5]^{2-}$ (**3b**) and $[W^{VI}(TMSI)Cl_5]^-$ (**3c**). Colour code: carbon (C, gray), chlorine (Cl, lime green), hydrogen (H, white), nitrogen (N, blue), silicon (Si, yellow), and tungsten (W, light blue).

S7 References

[1] I. M. Alecu, J. Zheng, Y. Zhao, D. G. Truhlar, *J. Chem. Theory Comput.* **2010**, *6*, 2872–2887.

[2] S. Fortier, J. L. Brown, N. Kaltsoyannis, G. Wu, T. W. Hayton, *Inorg. Chem.* **2012**, *51*, 1625–1633.

[3] A. V. Korolev, A. L. Rheingold, D. S. Williams, *Inorg. Chem.* **1997**, *36*, 2647–2655.

[4] M. P. Mehn, S. D. Brown, D. M. Jenkins, J. C. Peters, L. Que, *Inorg. Chem.* **2006**, *45*, 7417–7427.

[5] T. W. Hayton, J. M. Boncella, B. L. Scott, P. D. Palmer, E. R. Batista, P. J. Hay, *Science* **2005**, *310*, 1941–1943.
